# Podcasts for the Delivery of Medical Education and Remote Learning

**DOI:** 10.2196/29168

**Published:** 2021-08-27

**Authors:** Julliana Newman, Andrew Liew, Jon Bowles, Kelly Soady, Steven Inglis

**Affiliations:** 1 Gilead Sciences Melbourne Australia; 2 Oxford PharmaGenesis Melbourne Australia; 3 Oxford PharmaGenesis Oxford United Kingdom; 4 PharmaGenesis London London United Kingdom

**Keywords:** digital, hepatitis C virus, health care professionals, hepatology, HIV, continuous professional development, podcasts, remote learning, virology

## Abstract

Podcasts are increasingly being recognized as an effective platform to facilitate the continuous professional development (CPD) of health care professionals (HCPs). Compared with face-to-face meetings and other more traditional forms of CPD, podcasts allow for flexible learning and are less expensive to develop. Podcasts are at the cutting edge of digital education and can be an important element of a pharmaceutical company’s multichannel communications plan to improve HCP engagement and CPD in specific therapy areas. However, developing a successful podcast can have significant challenges. In this viewpoint paper, we provide our perspectives on medical podcasts as a medium for educating HCPs in the digital age. We describe our experience in developing an HIV-focused podcast for Australian HCPs, creating a series that has now expanded to other therapy areas in several countries. Practical considerations and unique challenges associated with industry-sponsored podcasts are outlined. Overall, we believe that the process of developing a podcast can be a challenging but rewarding experience, and CPD delivered via podcasting should be more routinely considered by pharmaceutical companies.

## Introduction

Podcasts have gained in popularity during the mid-2000s and are now widely available on the internet and on various on-demand channels [1]. Although many podcasts have been developed for entertainment, they are increasingly being used as a platform for medical education [2-5], and recent review articles have shown that a wide variety of medical podcasts covering numerous medical specialties are available [2,6,7]. Medical journals such as *The New England Journal of Medicine* [8] and *The Lancet* [9] are using podcasts to discuss contemporary topics in medicine, further supporting the idea that medical podcasts can supplement more traditional forms of learning, such as peer-reviewed journal articles.

This rise in the popularity of podcasts among the medical community is probably due to several factors. Podcasts allow for rapid dissemination of up-to-date data [10] and provide a range of perspectives to listeners (eg, patient perspectives). The availability of on-demand content helps listeners to learn on the go, which is especially important for busy health care professionals (HCPs) [10]. Unlike face-to-face lectures or congress presentations, podcasts avoid the need for and the expense of travel and give opportunities to HCPs from resource-limited settings to access new perspectives and data. Medical podcasts typically cater to a wide audience with different levels of knowledge (eg, clinicians, medical students, and other HCPs, nurses, pharmacists, and allied HCPs).

Medical podcasts can be grouped into two broad categories: industry-sponsored and general medical podcasts. Industry-sponsored podcasts are developed by (or in collaboration with) pharmaceutical companies, are typically developed exclusively for the continuing professional development (CPD) of HCPs, and focus on a particular therapy area or product. General medical podcasts are often developed by individuals or medical societies for a wider audience (eg, medical students or the general public, as well as HCPs) and cover a wide range of topics [2].

**Figure 1:**
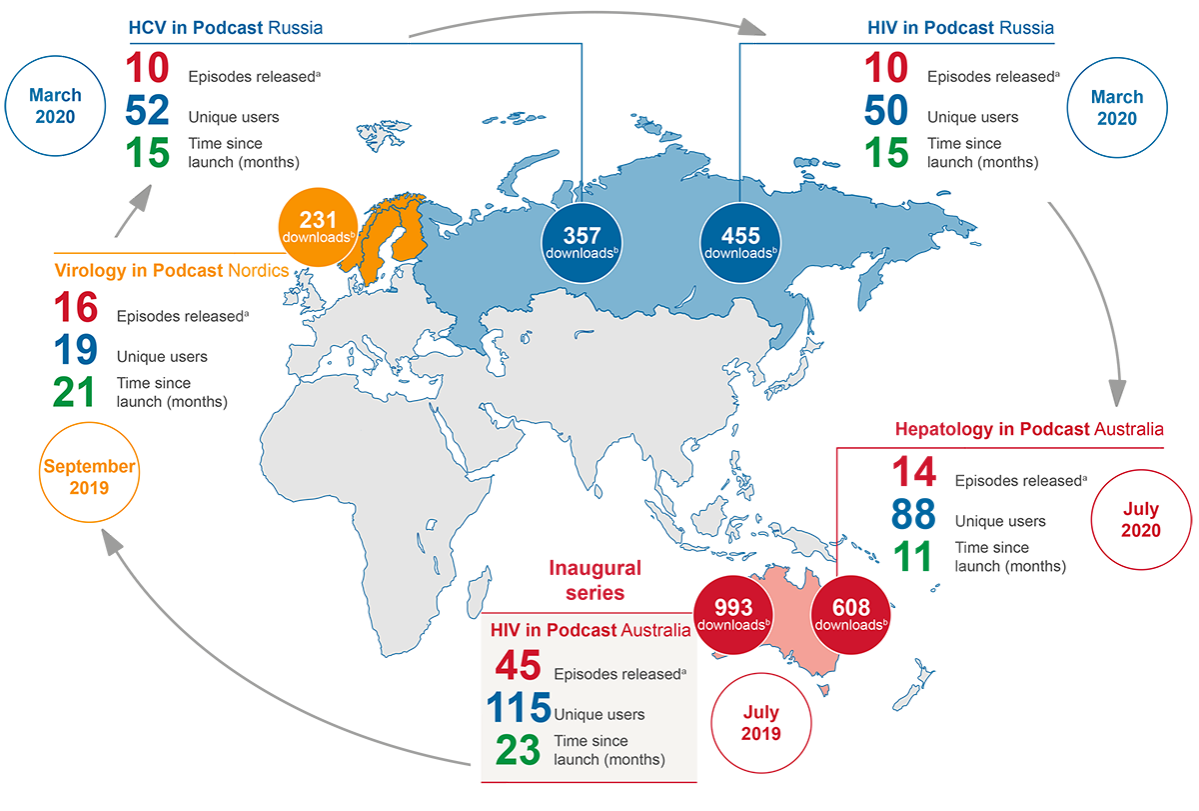
Overview of the *in Podcast* series and podcast metrics provided by the hosting platform. ^a^As of May 31, 2021. ^b^Audio file download to app or live stream across series.

We noted that there were very few digitally based CPD resources available circa 2018 for Australian HCPs who care for people living with HIV. In response, we launched *HIV in Podcast* Australia, a podcast that provides the latest insights into HIV care from leading experts. *HIV in Podcast* Australia was initially hosted on a web-based platform exclusive to HCPs [11] and in July 2019, we launched a dedicated app to house the podcast. Since then, the series has expanded to include additional therapy areas and regions, and now comprises the following: *HIV in Podcast* Australia, *Hepatology in Podcast* Australia, *Virology in Podcast* Nordics, *HCV in Podcast* Russia, and *HIV in Podcast* Russia (Figure 1). As appropriate, content was tailored to region-specific health challenges. Apart from the 2 Russian series, all podcasts were recorded in English.

In this paper, based on our own experience with the *in Podcast* program, we describe practical considerations and challenges associated with developing, distributing, and measuring the success of industry-sponsored podcasts. Several guides on creating effective general medical podcasts have been published [12-15]. However, to the best of our knowledge, there are no publications that discuss the unique challenges associated with developing industry-sponsored podcasts. Overcoming some of these challenges could contribute to an increase in the use of this CPD tool for HCPs.

## Practical Considerations for Industry-Sponsored Podcasts

To develop high-quality and engaging medical podcasts, a few basic elements are implicit. In short, these include the recruitment of a suitably qualified host with medical training and therapy area experience, ideally with medical journalism experience. Equally, interviewees should be regional or international experts in their field and be able to discuss contemporary topics concisely. Podcasts should be “bite-sized,” with recommended lengths of 10-20 minutes [3], and recorded and produced by professional sound engineers.

In our experience, development of industry-sponsored podcasts often present other unique challenges and considerations, which we describe in further detail below.

### Podcast Hosting Platforms

Podcast episodes need to be hosted on an appropriate digital platform for access by listeners [12]. A myriad of hosting options are available (eg, custom-built websites or apps and paid and free platforms) [12], and choosing a platform will depend on budgets, compliance requirements, and timelines. An ideal hosting platform should be secure and easy to use, provide reliable and standardized metrics, and be adaptable for local and regional regulatory requirements regarding distribution of content to HCPs. In general, access to podcast episodes can be achieved via the webpage of the hosting platform or a dedicated mobile app. Such apps should be easy to download, have intuitive interfaces, be compatible with both Apple and Android operating systems, and allow download and storage of episodes for offline listening. Based on these considerations, we chose a specialist podcast hosting platform [16] for the *in Podcast* series that is free of charge to the end user, with an optional associated app to provide HCPs with the convenience of accessing content via their mobile device. The app is available in multiple languages (to mirror the podcasts). It is important to note that developing such an app requires significant investment, likely including partnering with companies or individuals with app development expertise.

### Regulatory Considerations

A challenge encountered with industry-sponsored podcasts is ensuring that the discussions related to medications or clinical practice are in line with the approved indication for that region, fair, balanced, and consistent with local and regional guidelines governing communication with HCPs. For example, guests might describe treatments that are not approved in the country where the podcast is released, or they might discuss data from studies of unlicensed uses for approved agents; thus, content could be deemed promotional or noncompliant with local guidelines [17,18]. Medical writers, together with regulatory personnel of the sponsor company, play an important role in managing content development. In the *in Podcast* program, the medical writing team were involved in all aspects of episode development, including podcast recording sessions, to ensure that discussions between the podcast host and guests were accurate and reflected the approved indications for any treatment discussed (“on label”). The medical writing team were also responsible for postrecording activities such as transcribing the recording and ensuring that all scientific claims were supported by current evidence from peer-reviewed journal papers or government guidelines, with a reference list available for every podcast episode. We believe that medical writers play a crucial role, in partnership with the regulatory team, in ensuring that all podcast content is fair and balanced.

The *in Podcast* series has been launched across different regions; therefore, we had to ensure compliance with regulatory and privacy requirements for each of the relevant countries. The introduction of the European Union General Data Protection Regulation (GDPR) in 2018 implied ensuring the protection of personal data, privacy, and consent of podcast users. To achieve this, we engaged medical, regulatory, business compliance, and legal stakeholders in the region and therapy area when planning each series. Some hosting platforms collect personal data (eg, email addresses) for metrics analysis, so it is important to utilize a GDPR-compliant hosting platform.

**Figure 2:**
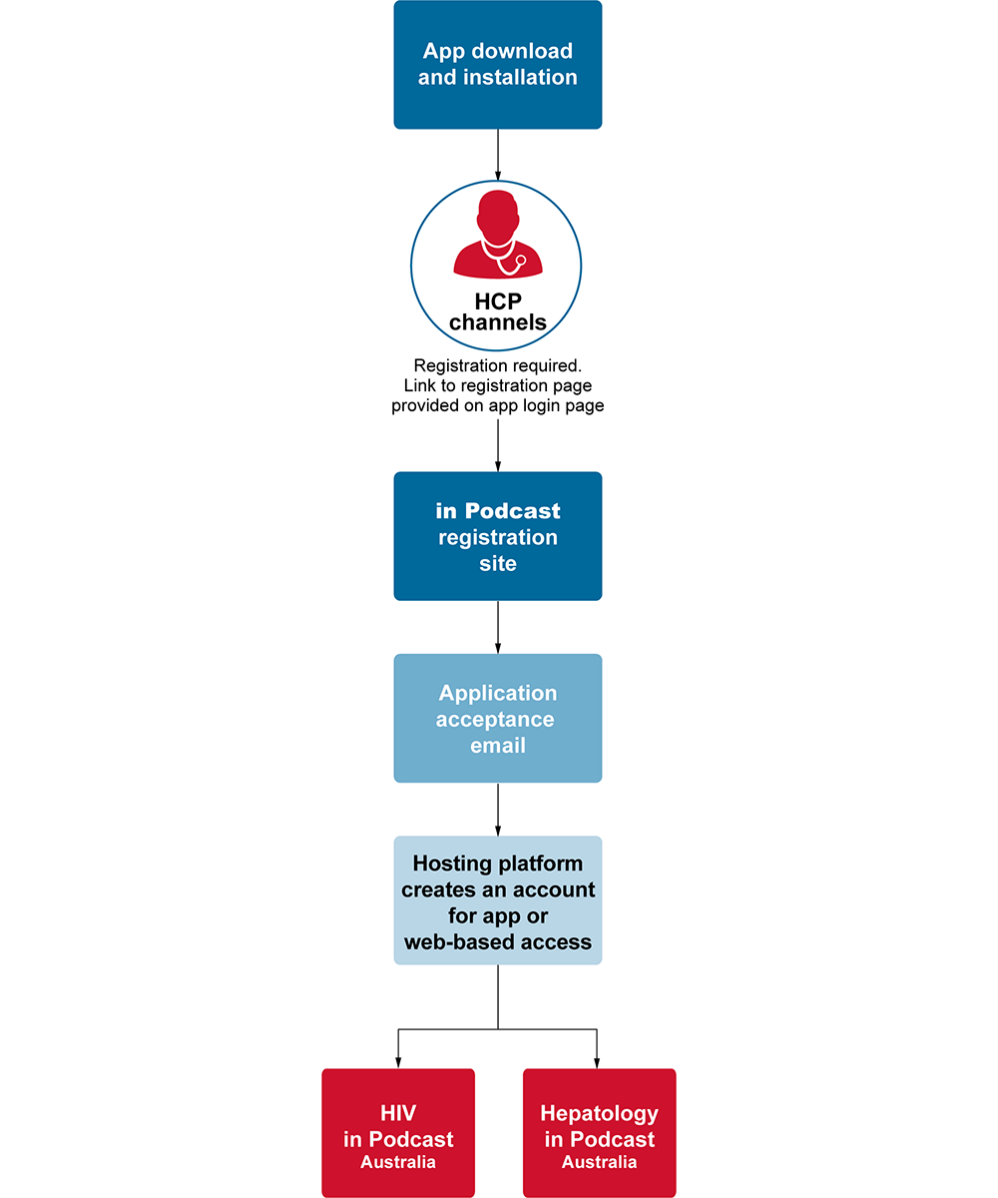
Podcast registration flow for Australian HCPs accessing *HIV in Podcast* Australia or *Hepatology in Podcast* Australia episodes. Channel access is determined by geographical location upon registration, so that Australian HCPs will only have access to Australian channels. HCP: health care professional.

In some countries, discussion or promotion of prescription medicines is only permitted among HCPs. As such, industry-sponsored podcast topics may only be appropriate for an HCP audience (eg, content that could be perceived as promotion of a pharmaceutical product to a non-HCP audience). To ensure that only HCPs could access the content, we created a registration webpage that allowed us to verify a user’s HCP status via their medical/professional registration numbers, or enabled the HCP to self-verify their status, depending on local regulatory requirements. Once a user’s HCP status is verified, they are sent detailed instructions on how to download the podcasting app, create a personal podcast account, and access episodes via the app or a web browser. A summary of the user registration process we used is shown in Figure 2. Because HCPs register through a country-specific registration page, they are given access to content available in their region only. This system enables 1 platform and mobile app to be used across multiple regions.

### Accreditation of Podcasts

The credibility of industry-sponsored podcasts can be enhanced through accreditation by a professional body (a medical society or college), thereby allowing HCPs to use the podcasts for CPD. Medical practitioners registered with the Medical Board of Australia, for example, need to complete a minimum number of CPD hours that are relevant to their practice [19]. Accreditation of podcasts may thus help to increase their uptake by the target audience. To gain accreditation, podcast transcripts and supporting references for each episode of *HIV in Podcast* Australia were reviewed by the Australasian Society of HIV Medicine [20]. Users were encouraged to self-report their listening activity to Australasian Society of HIV Medicine to claim CPD points and maintain their registration as prescribers of HIV treatment. It is important to note that the role of CPD points in continuing professional medical education and accreditation requirements varies across regions. The medical writing team plays an important role in ensuring that all podcast materials meet the accreditation standards of the relevant accrediting society.

### Audience Recruitment

Recruiting an appropriate and sizable audience can be challenging. Promoting general medical podcasts typically involves the use of social media platforms (eg, blogs, Facebook, and Twitter) [13] or joining a podcast network [21]. However, if applied to industry-sponsored podcasts, this approach may be considered an untargeted promotional activity and tends to be avoided by pharmaceutical sponsors. Instead, we employed several focused strategies to increase the awareness of the podcasts among HCPs, including the use of hard copy and electronic mailouts to HCPs on subscriber lists provided by industry partners, publishing printed advertisements in peer-reviewed journals or scientific magazines, and conducting promotional activities at scientific meetings (setting up booths, handing out flyers, and conducting in-person registrations). We found that electronic mailouts reach a wider, more targeted audience than the other methods but may require the use of external platforms (eg, Mailchimp) or third parties (eg, advertising or event management agencies who have access to lists of potential subscribers). We found that an advantage of promoting podcasts at scientific meetings was that it provided us with opportunities to gain valuable feedback on the podcasts. We recommend that multichannel promotional strategies be used, if possible, to maximize podcast uptake.

### Measuring the “Success” of Podcasts

Regularly measuring the uptake of podcasts by HCPs is essential for evaluating the performance of a podcast and can be an indicator of the success of the strategies used to attract listeners. The number of downloads is one of the most frequently used digital performance metrics [3], although other metrics are available (eg, the number of podcast users) [22]. At the time of writing (May 31, 2021), *HIV in Podcast* Australia had achieved the largest number of total downloads and unique users (993 downloads; 115 unique users) followed by *Hepatology in Podcast* Australia (608 downloads; 88 unique users; Figure 1). In Europe and Russia, *HIV in Podcast* Russia achieved the largest number of total downloads (455 downloads), whereas *HCV in Podcast* Russia had the highest number of unique users (52 users). Relative to the small number of unique users, the large number of downloads suggests that users likely downloaded multiple podcast episodes, which indicates continued engagement with content over time. In our experience, the number of downloads can be influenced by various factors including therapy area, size of the target audience, geographical region, and how readily the target audience can access the podcast. This makes direct comparisons of podcast performance difficult. For example, average monthly downloads for the *in Podcast* series, housed on our app in which HCPs must register to gain access, were between 56 downloads per month for *Hepatology in Podcast* Australia and 11 downloads per month for *Virology in Podcast* Nordics (Figure 1). In contrast, publicly available medical podcasts have been reported to achieve between 217 and 10,000 downloads per month [10,23,24]. Comparisons with other industry-sponsored podcasts are challenging owing to a lack of published data.

Another important consideration is that download metrics provided by GDPR-compliant hosting platforms contain anonymized user identifications. This makes it difficult to profile podcast users by HCP type (eg, proportion of physicians vs nurses), which may be of specific interest to the sponsor. Additional qualitative measures of success may therefore add value. For example, short surveys can be included in electronic mailouts or conducted via face-to-face interviews with the target audience [10]. These qualitative data can be used to understand if, and the extent to which, listeners are enjoying the series and whether it may be changing their clinical practice. Additionally, podcast performance metrics should be codeveloped with industry partners and realistic goals set for measuring “success” (eg, a prespecified proportion of licensed prescribers within a therapy area in 1 country). We believe that combining both quantitative and qualitative metrics provides more holistic insights into the overall performance of the podcast.

## Viewpoint

There is a growing interest from the pharmaceutical industry to invest in digital innovation, such as podcasts, to increase awareness of diseases, clinical practice updates, and available therapies. At present, most medical podcasts have been created by individuals and academic societies rather than pharmaceutical companies. Medical podcasts are an important medium for delivering specific and targeted CPD to HCPs and could be considered more routinely by the pharmaceutical industry as part of multichannel communication plans to improve HCP engagement and education. However, very little published information is available on the considerations and challenges associated with creating industry-sponsored podcasts.

In our opinion, one key challenge is the limitation around granular metrics data on industry-sponsored podcasts, making it difficult to demonstrate a return on investment. Close collaboration with industry partners will be needed to define the target audience and to help ensure that meaningful metrics are collected. The inclusion of qualitative measures (eg, user surveys) is also recommended to determine if podcast uptake is associated with changes in clinical practice.

The requirement for users to register and be verified as HCPs before being able to access the podcasts may also be a significant “digital barrier” to the uptake of industry-sponsored podcasts. We suspect that multistep registration processes (Figure 2) and the need to create a personal podcast account may act as a deterrent for busy HCPs to quickly access podcast content. However, these are necessary steps from an industry compliance perspective in many jurisdictions. Possible options for overcoming this challenge could include setting up preregistrations and involving medical science liaison officers of companies to help users with the registration process. If regulations allow, creating podcast apps that support single-click access from an invitation email for HCPs who have been preregistered and verified could reduce the steps required for authentication and prevent possible “password fatigue,” which may result from users needing to manage multiple passwords and accounts.

Overcoming these barriers could help increase the use of this flexible and versatile educational platform among the medical community. Indeed, recent innovations have highlighted that podcasting will be an important medium for educating HCPs in the future. For example, several Adis-affiliated journals allow the publication of peer-reviewed podcast articles on clinically-relevant topics (eg, conference data, treatment innovations, and expert opinions on drugs and disease therapies) [25]. We believe that the challenges and potential solutions we have outlined are not only relevant for podcasting but also broadly applicable to the various forms of industry-sponsored digital education (eg, webinars and education modules) that are increasingly being used.

## Conclusions

Podcasting is a novel method for delivering medical education and can be a suitable alternative to traditional face-to-face and print-based education and incorporated into CPD programs. Careful planning is needed to ensure the success of industry-sponsored podcasts. With further uptake and optimization of this form of medical education, industry-sponsored podcasts may play an important role in shaping change in clinical practice and improving patient care in the future.

## Abbreviations

CPD: continuing professional development
GDPR: General Data Protection Regulation
HCP: health care professional
